# Maternal–Fetal Transmission of *Cryptococcus gattii* in Harbor Porpoise

**DOI:** 10.3201/eid1702.101232

**Published:** 2011-02

**Authors:** Stephanie A. Norman, Stephen Raverty, Erin Zabek, Sandra Etheridge, John K.B. Ford, Linda M.N. Hoang, Muhammad Morshed

**Affiliations:** Author affiliations: Marine-Med: Marine Research, Epidemiology, and Veterinary Medicine, Bothell, Washington, USA (S.A. Norman);; Central Puget Sound Marine Mammal Stranding Network, Greenbank, Washington, USA (S.A. Norman);; British Columbia Ministry of Agriculture and Lands, Abbotsford, British Columbia, Canada (S. Raverty, E. Zabek, S. Etheridge);; Fisheries and Oceans, Canada, Nanaimo, British Columbia, Canada (J.K.B. Ford);; British Columbia Center for Disease Control, Vancouver, British Columbia, Canada (L.M.N. Hoang, M. Morshed);; University of British Columbia, Vancouver (L.M.N. Hoang, M. Morshed)

**Keywords:** Cryptococcus gattii, fungi, maternal–fetal transmission, harbor porpoise, Phocoena phocoena, Pacific Ocean, letter

**To the Editor:** We report maternal–fetal transmission of *Cryptococcus gattii* and death in a wild porpoise. *Cryptococcus neoformans* and *C. gattii* are 2 environmental, encapsulated yeasts that cause invasive, potentially life-threatening infections in humans and animals (*1*). *C. neoformans* causes disease in immunocompromised hosts, and *C. gattii* is also pathogenic in immunocompetent hosts (*2*). Since 1999, cryptococcosis caused by *C. gattii* has appeared on southern Vancouver Island (British Columbia, Canada) and nearby surrounding areas (*2**,**3*). Spread beyond Vancouver Island has been documented along the Pacific Northwest Coast, but the mechanism remains undetermined (*4*).

A pregnant, dead, stranded, harbor porpoise (*Phocoena phocoena*) was reported on February 22, 2007, on western Whidbey Island, in Puget Sound, Washington State (48.2833°N, 122.7283°W). The carcass was iced and necropsy was performed on February 24. Sampled tissues from the adult and fetus were divided: half fixed in 10% formalin for histopathologic analysis, and half frozen for ancillary studies.

For histologic analysis, tissues were embedded in paraffin, sectioned to 3–5 μm, and stained with hematoxylin and eosin. Selected sections were stained with mucicarmine. The adult porpoise (length 177 cm, weight ≈57.7 kg) was in poor condition (reduced blubber layer). Both lungs were exposed, extensively scavenged, firm, and nodular; a sectioned surface exuded clear to slightly opaque gelatinous to mucinous discharge. Mediastinal lymph nodes were grossly enlarged, multinodular, and firm with large numbers of yeasts visible by microscopy (online Figure A1 panel A, www.cdc.gov/EID/content/17/2/304-appF.htm). The first stomach chamber contained two 3.5 cm × 2.5 cm raised, centrally umbilicated ulcers and several embedded anisakid nematodes. The uterus was gravid in the right horn with a mid-term fetus. No other gross lesions were identified. Microscopically, the lung lesions correlated with granulomatous to pyogranulomatous infiltrates, often with a myriad of yeasts.

The male fetus (length 30 cm, weight 2.4 kg), was examined separately at a different facility than the dam. It appeared grossly normal externally and was at a gestation of ≈5–6 months. Mediastinal lymph nodes had mild granulomatous inflammation and contained numerous yeasts morphologically consistent with *Cryptococcus* spp. (Figure A1, panel B). The lymph nodes were partially replaced with intracellular and extracellular mulilobulated yeast aggregates (length 8–20 μm) with pale eosinophilic central regions and a thin refractile wall peripherally bound by a 5-μm nonstaining capsule. Around the periphery of these aggregates, there were small numbers of macrophages and lymphocytes and fewer neutrophils. Specific staining showed a prominent mucicarminophilic capsule consistent with *Cryptococcus* spp.

Yeasts were found in the amniotic fluid and interspersed within the chorioallantoic villi and submucosal vasculature of the placenta. Mild multifocal nonsuppurative myocarditis was detected. However, no yeasts were seen in inflamed areas. There were no overt lesions in the remaining organs.

Maternal and fetal tissues were cultured for fungi, and diagnosis was based on Gram stain (budding yeast-like cells), India ink stain (positive for encapsulated cells), hydrolysis of urea (positive), and final confirmation by using API 20C Aux V3.0 (bioMérieux, Marcy l’Etoile, France). Canavanine-glycine-bromthymol blue agar was used to differentiate between *C. gattii* and *C. neoformans* (*5*). Molecular typing by restriction fragment length polymorphism was used to definitively speciate and subtype *C. gattii* (*6*). Fungal culture showed heavy growth of *Cryptococcus* spp. from the dam (lungs, mediastinal lymph nodes, and placenta) and fetus (mediastinal lymph nodes). Genotyping of primary isolates identified VGIIa *C*. *gattii* in both animals. Test results for enteric pathogens, intestinal nematodes, morbillivirus, and *Brucella* spp. were negative.

Fetal infection was most likely hematogenous, disseminated from a primary maternal pulmonary source to the uterus and subsequently to placental vasculature and internal fetal tissues. Infection by aspiration or ingestion of contaminated amniotic fluid was also possible. Although close evaluation of the lung did not show any discernible yeasts, the organism may have been present in an area other than that sectioned.

During 1998–2007, ≈450 harbor and Dall’s porpoises (*Phocoenoides dalli*) and Pacific white-sided dolphins (*Lagenorhynchus obliquidens*) along the Pacific Northwest Coast were recovered and subjected to necropsy. Disseminated cryptococcosis caused by *C. gattii* since 2000 was diagnosed in 15 harbor porpoises, 10 Dall’s porpoises, 2 adult Pacific white-sided dolphins, and 3 unrecorded species (10 females, 15 males, and 5 unknown sex; 24 adults, 4 juveniles, 1 fetus, and 1 undocumented age; S. Raverty, unpub. data).

Wild porpoises in the Pacific Northwest Region, being near shore inhabitants in waters surrounding Vancouver Island, may come into contact with air containing *C. gattii* at the air–water interface or ingest seawater containing yeasts while feeding (*7*). Their proximity to a habitat containing Coastal Douglas fir (*Pseudotsuga menziesii*) and Western hemlock (*Tsuga heterophylla*) may play a role in the epidemiology of *C. gattii* because these trees have been associated with cases of *C. gattii* (*8*). Cryptococcal infection during pregnancy has been reported in humans and horses (*9**,**10*).

This fetal case of cryptococcosis may have major human and animal health implications. Further studies should be undertaken to assess possible fetal involvement, identify infections in pregnant females, and provide information on risk reduction and improving diagnosis and treatment.

## Figures and Tables

**Figure A1:**
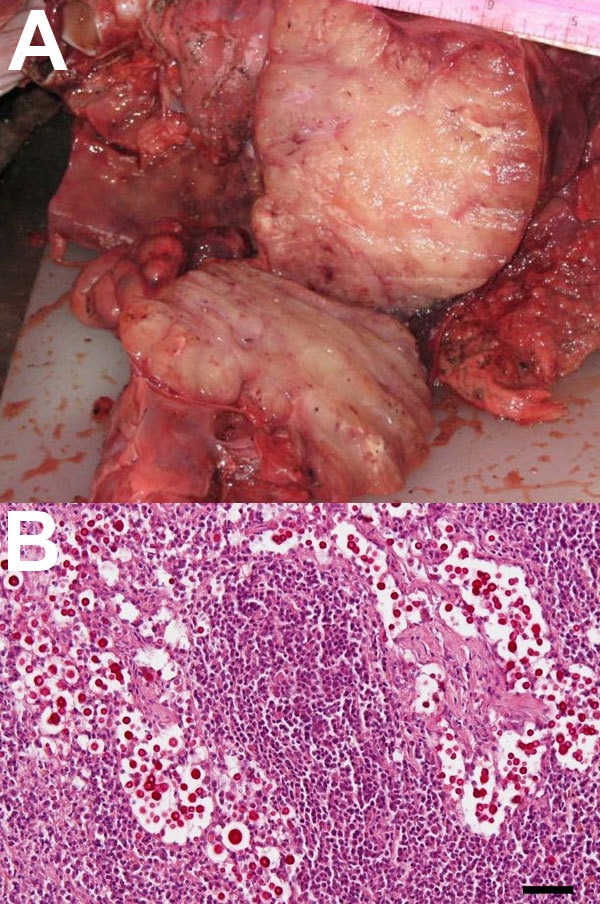
A) Enlarged mediastinal lymph nodes of a stranded, pregnant, harbor porpoise (Phocoena phocoena) infected with Cryptococcus gattii that was transmitted to its fetus. B) Mucicarmine–stained sections of fetal mediastinal lymph node, showing C. gattii extracellular yeast aggregates (original magnification ×20). Scale bar = 50 μm.
